# Study on the Diagnosis of Gastric Cancer by Magnetic Beads Extraction and Mass Spectrometry

**DOI:** 10.1155/2020/2743060

**Published:** 2020-08-05

**Authors:** Ning Zhu, Xiaoliang Xing, Limei Cao, Yingjun Zhang, Ti Zhang, Zhen Li, Fen Zou, Qing Li

**Affiliations:** ^1^Hunan Provincial Key Laboratory for Synthetic Biology of Traditional Chinese Medicine, School of Public Health and Laboratory Medicine, Hunan University of Medicine, Huaihua, 418000 Hunan, China; ^2^Chenzhou No.1 People's Hospital, Chenzhou, 423000 Hunan, China; ^3^South China University of Technology, Guangzhou, 510000 Guangdong, China

## Abstract

**Objective:**

This study constructed a model for the early diagnosis of gastric cancer by comparing the serum peptides profiles of patients with advanced gastric cancer and healthy people. And that model may be the potential to be applied for the efficacy evaluation and recurrence monitoring in gastric cancer.

**Methods:**

Serums of 30 healthy people and 30 advanced gastric cancer patients were matched by age and gender were collected. The serum peptide spectrum was obtained by MB-WCX concentration and MALDI-TOF MS analysis. Based on the analysis of the efficiency of differential peptides in the diagnosis of gastric cancer, we first established a model for the diagnosis of gastric cancer based on differential peptides and then carried out external verification. The diagnostic reliability of this model was further tested by compared with carcinoembryonic antigen (CEA) and carbohydrate antigen 19-9 (CA19-9).

**Results:**

In this present study, we found the expression of two peptide peaks with a molecular weight of 2863 Da and 2953 Da were significantly increased in gastric cancer serum, while the expression of two peptide peaks with a molecular weight of 1945 Da and 2082 Da were significantly decreased. Depending on the characteristics of peptide expression, we constructed a diagnostic model, we compared the sensitivity and specificity of the model established by 2953 Da/1945 Da, and found this model is significantly higher than CEA and CA19-9.

**Conclusion:**

There were some differences in serum peptides profiles between patients with advanced gastric cancer and healthy people. The serum peptide diagnostic models based on 2953 Da and 1945 Da have high diagnostic efficiency for advanced gastric cancer. Our result indicated that this model was well worth further validation for clinical diagnosis.

## 1. Introduction

Gastric cancer (GC) is the fifth most common cancer and is the third leading cause of death worldwide [1]. GC mostly occurs to the 50-70 age groups, and the overall incidence of GC has declined in the last decade, and the male-to-female average ratio is 1.79 [2]. Despite remarkable advances in comprehensive treatment approaches, the prognosis of GC patients remains poor, with 5-year overall survival ranging between 15 and 35% [3]. Therefore, early diagnosis plays an important role in improving treatment effects and survival rates.

Finding tumor markers with high sensitivity and specificity is an effective method to solve the problem of early tumor diagnosis. Serum peptides refer to all small molecular protein fragments and peptides with molecular weight less than 50 kDa in serum. Accumulating studies indicated that many low molecular weight tumor-derived proteins and peptides could be released into the peripheral blood circulation from tumor tissues. In 2002, Petricoin et al. found that ovarian cancer patients and healthy people can be distinguished by detecting peptides in serum [4]. And both the sensitivity and specificity were significantly higher than CA125 [4]. This is the first study of serum peptide as a tumor marker. In recent years, with the rapid development of biomass spectrometry technology, serum peptides, which used to be considered as biological waste, has become a hot spot in screening tumor blood markers [5, 6]. Therefore, the value of serum peptides in tumor diagnosis has been gradually recognized.

In this study, magnetic bead solid-phase extraction combined with mass spectrometry was selected to study serum peptides in gastric cancer which was based on the previous studies of serum peptides. Through comparison of serum peptides profiles, the peptide expression of 2863 Da and 2953 Da were significantly increased, and 1945 Da and 2082 Da were significantly decreased. The sensitivity and specificity of the diagnostic model established based on 2953 Da and 1945 Da were significantly higher than CEA and CA19-9. This study provides an experimental basis for the establishment of a serological diagnosis method of gastric cancer based on serum peptides.

## 2. Materials and Methods

### 2.1. Object

Advanced gastric cancer group, 30 serum samples of patients with advanced gastric cancer from the second affiliated hospital of the University of South China, and 30 serum samples of healthy volunteers were collected from the physical examination center of the second affiliated hospital of the University of South China (Table 1). The present study protocol was approved by the Ethics and Research Committees of Hunan University of Medicine (Huaihua, China) and was conducted in accordance with the principles outlined in The Declaration of Helsinki.

30 patients with gastric cancer and 30 patients with physical examination were randomly divided into training groups (including 20 patients with gastric cancer and 20 patients with physical examination) and verification group (including 10 patients with gastric cancer and 10 patients with physical examination) according to a ratio of 3 : 1. The training group was used to establish the gastric cancer diagnosis model, and the validation group was used to evaluate the diagnostic efficacy of the model.

### 2.2. Reagents and Apparatus

The Quantitative determination kit for CEA and CA19-9 were from Roche Company (US), MB-WCX was from Bruker Company (Germany), Standard proteins and peptides were from Bruker Company (Germany), and MALDI-TOF MS system was from Bruker Company (Germany).

### 2.3. WCX Magnetic Bead Processing

Take out a tube of weakly cation magnetic bead suspension, shake it up and down, and completely mix the magnetic bead suspension for 1 min; Take out 1 *μ*L magnetic bead combination buffer and add it into 200 *μ*L tube, then add 10 *μ*L magnetic bead to the sample tube, suck and mix it up and down, and avoid foaming; Add 8 *μ*L serum to the sample tube, mix it completely, and avoid foaming; Stand at room temperature for 5 min, and separate the magnetic beads from the suspension; Add 100 *μ*L magnetic bead cleaning buffer into the sample tube; Repeatedly move the sample tube 10 times of the two adjacent holes before and after the magnetic bead separator, and separate magnetic beads from the suspended liquid; Take the sample tube from the magnetic bead separator, add 5 *μ*L magnetic bead eluent buffer into the sample tube, mix the magnetic beads attached to the wall, and suck and beat them for 10 times repeatedly, avoid foaming and incubate at room temperature for 2 min; Add 5 *μ*L stable buffer solution to 0.5 mL new sample tube; The sample tube was put into a magnetic bead separator, and the magnetic bead stuck to the wall for 2 min; After the magnetic bead was fully separated from the suspended liquid, the supernatant was transferred into the 0.5 mL sample tube with stable buffer solution, which was carefully mixed on the ice.

### 2.4. MALDI-TOF Mass Spectrometry

Wash and dry the 384 point stainless steel target; the positions of sample spots and external punctuation marks are prearranged on the sample table to ensure that each sample spot is adjacent to an external punctuation mark; 1 *μ*L sample (magnetic bead eluent) was arranged to the corresponding location of the stainless steel target; 0.5 *μ*L standard protein and peptide was arranged to the same location; 1 *μ*L saturated HCCA matrix (freshly prepared with 0.1% TFA solution) after the sample point is dried; dry to completely dry; the sample target was sent to mass spectrometry for analysis within 2 h after drying; external calibration shall be carried out with the adjacent external standard before analyzing the sample each time; mass spectrometry parameter setting: linear mode, first-order mass spectrometry, acquisition range: 0-10000da, 60% laser energy, 100 shots × 10 stack.

### 2.5. Sample Analysis

5 mL blood was collected in the early morning and reversed slightly for 3 times, and then stand for 1 h at room temperature, and centrifugation at ×1600 g, 10 min, 4°C. Carefully transfer the upper layer of serum to 1.5 mL tube on ice and store at -80°C. Add 10 *μ*L magnetic bead binding buffer, 10 *μ*L magnetic bead, and 8 *μ*L serum to 200 L sample tube and mix. After separation, add 100 *μ*L magnetic bead cleaning buffer and repeat the previous step 2 times. Add 5 *μ*L magnetic bead elution buffer, after separation, transfer the supernatant into a new 0.5 *μ*L sample tube, add 5 *μ*L magnetic bead stability buffer and suck and mix. 1 *μ*L standard material was mixed with 10 *μ*L substrate, and then 1 *μ*L mixed liquid was selected to be the anchorage chip target standard. The sample was analyzed by MALDI-TOF MS system.

### 2.6. Statistical Analysis

FlexAnalysis software was used to analyze the peak number and peak strength data of the original mass spectrogram of each sample (S/N > 5). Smoothing, baseline deduction, and recalibration of the mass spectrometry were performed using ClinProTools. ClinProTools software is used for peak calculation (peak strength), peak statistics, ROC (Receiver Operating Characteristic) curve Analysis, Principle Component Analysis (PCA). SPSS 15.0 software is used for Statistical Analysis. Chi-square test is used for counting data, and *t*-test is used for measurement data.

## 3. Results

### 3.1. Differential Analysis of Serum Peptides Expression Profile between Healthy and Gastric Cancer Patients

**Figure 1:**
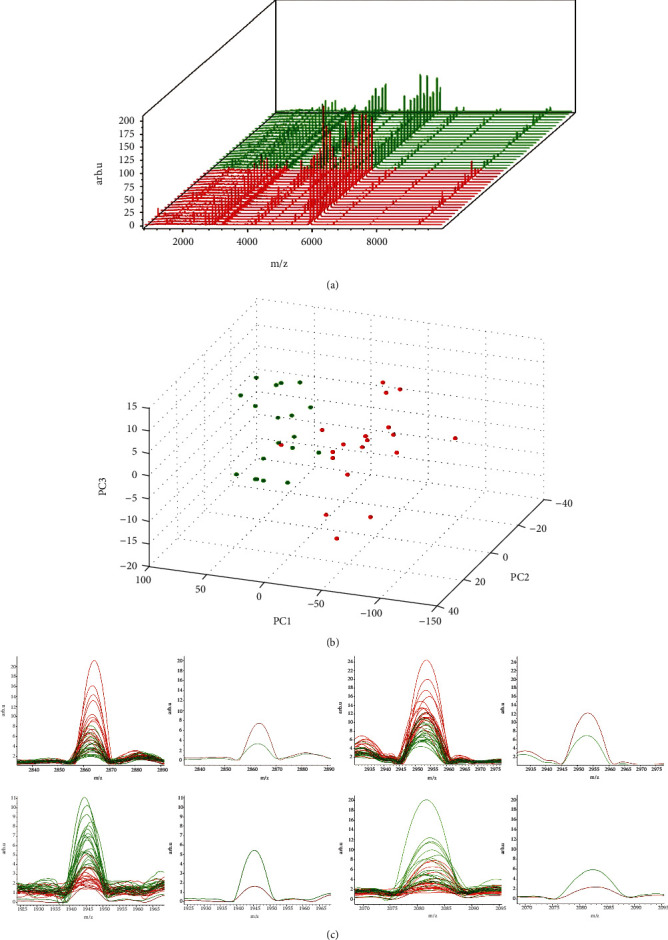
Differential analysis of serum peptides expression profile between healthy and gastric cancer patients. **(**a) The serum peptide expression profiles of 20 healthy patients and 20 gastric cancer patients were summarized (The horizontal coordinate is mass/charge (m/z), reflecting molecular weight. The ordinate is the sample number: red is the serum sample group of gastric cancer, green is the serum sample group of healthy people). (b) PCA plots of gastric cancer group and healthy group (Red: gastric cancer group; Green: health group). (c) Statistics of 4 differential peptide peaks (left: peptide peaks of all 40 samples; in average peak diagram of the two groups; right: Box diagram. Gastric cancer is red, healthy is green).

The serums of all 60 samples were extracted by MB-WCX and analyzed by MALDI-TOF MS to obtain high-quality peptide peak spectra. ClinPro 2.2 software was used to analyze the peak spectra of serum polypeptides in 20 healthy control and 20 gastric cancer patients. The signal-to-noise ratio (S/N) was set as >5, and the uncertain peptide peaks with small signal-to-noise were filtered out (The peak range of peptide mass spectrum was 0-10000 Da). 122 peptide peaks were obtained (Figure 1(a), Supplementary Table 1). All 122 peptide peak data were carried on normalized and compared analysis depend to the total number of ions. The Principal component analysis result (PCA) showed that the two groups could be separated, which indicated that there were many differences in the serum peptide expression between healthy control and gastric cancer patients (Figure 1(b)).

After calculation and statistics by ClinPro software, 4 peptide peaks (*p* < 10^−6^) with significant differences between the two groups were screened, No. 26 (2863 Da) and No. 29 (2953 Da) was increased while No. 16 (1945 Da) and No. 17 (2082 Da) was decreased (Figure 1(c)).

### 3.2. Establishment of Serum Peptides Diagnostic Model

**Figure 2:**
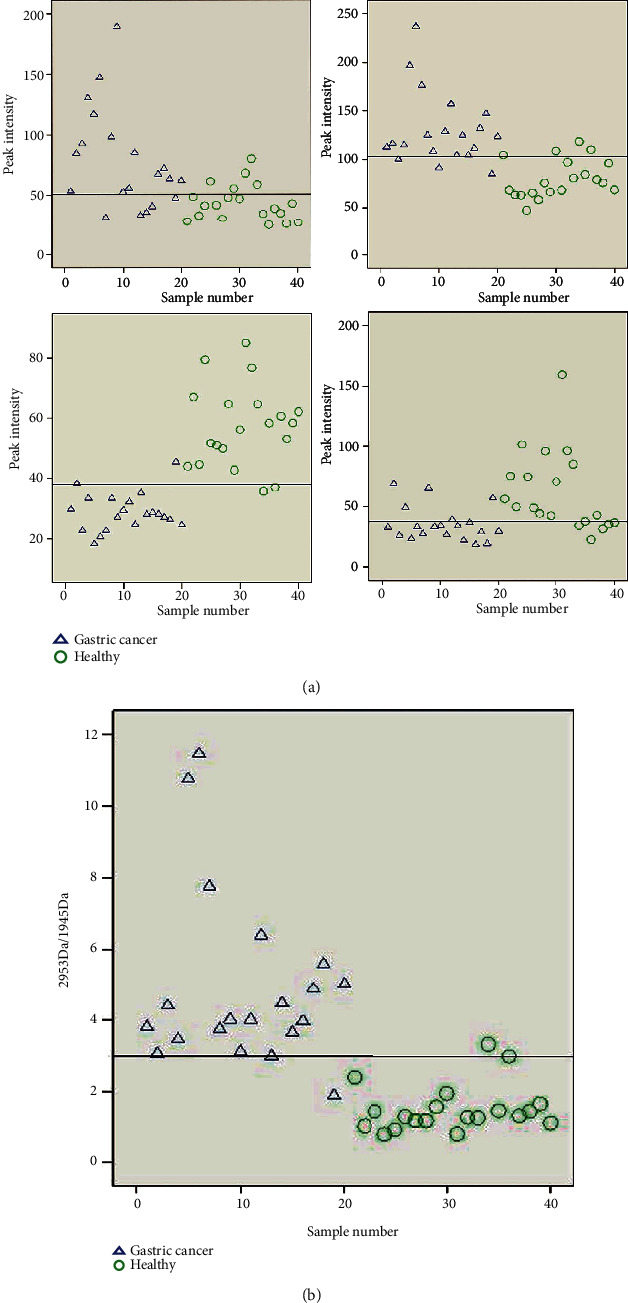
Establishment of serum peptides diagnostic model based on No.29 (2953 Da)/No.16 (1945 Da). (a) Diagnostic efficiency analysis of 4 differential peptide peaks (the horizontal line in the figure is the median numerical line). (b) The diagnostic effect of gastric cancer based on the ration of No.29 (2953 Da)/No.16 (1945 Da).

The efficiency of each peptide as a diagnostic marker in the diagnosis of gastric cancer was analyzed separately. The results showed that the sensitivity and specificity of No. 26 (2863 Da), No. 29 (2953 Da), No. 16 (1945 Da), and No. 17 (2082 Da) were 75.0%, 85.0%, 90.0%, and 75.0%, respectively (Figure 2(a)).

To improve the sensitivity and specificity of serum peptides in the diagnosis, we combined two points (No. 29 (2953 Da) and No. 16 (1945 Da)) with a high diagnostic rate for joint analysis. The specific manner is that the diagnosis of gastric cancer was calculated by the ratio of No. 29 (2953 Da)/No. 16 (1945 Da). The higher the ratio, the more prone the sample was to gastric cancer. The value 2.98 of No. 29 (2953 Da)/No. 19 (1945 Da) was taken as the reference index. The result of the sensitivity and specificity were close to 95.0% (19/20) (Figure 2(b)).

### 3.3. Validation of Serum Peptides Diagnostic Model in Gastric Cancer Patients

To further verify the reliability of the diagnostic gastric cancer model, the other 20 samples (10 healthy control and 10 gastric cancer patients) were used for external validation. The results showed that only 1 case was a false positive and false negative, with specificity and sensitivity of 90.0% and 90.0%, respectively, the total error rate was 10.0% (2/20) (Table 2).

### 3.4. Compared Analysis of Serum Peptides Model with CEA and CA19-9

The diagnosis result of CEA showed that the number of healthy control with the CEA value exceeds the reference was 5, and the average value of CEA for 30 healthy controls was 1.8 ng/mL. The number of gastric cancer patients with the CEA value exceeds the reference was 22, and the average value of CEA for 30 gastric cancer patients was 23.3 ng/mL. These results indicated that the positive rate and average value of CEA in gastric cancer patients were significantly higher than those in healthy controls (Table 3).

The diagnosis result of CA19-9 showed that the number of healthy control with the CA19-9 value exceeds the reference was 6, and the average value of CA19-9 for 30 healthy controls was 11.2 U/mL. The number of gastric cancer patients with the CA19-9 value exceeds the reference was 19, and the average value of CA19-9 for 30 gastric cancer patients was 53.7 U/mL. These results indicated that the positive rate and average value of CA19-9 in gastric cancer patients were significantly higher than those in healthy controls (Table 4).

All of these results indicated that the sensitivity and specificity of CEA in the diagnosis of gastric cancer were 73.3% (22/30) and 83.3% (25/30), respectively. The sensitivity and specificity of serum CA19-9 in the diagnosis of gastric cancer were 63.3% (19/30) and 80.0% (24/30), respectively. But the sensitivity and specificity of serum peptides model we constructed in the diagnosis of gastric cancer were 90.0% (27/30) and 90.0% (27/30). The diagnostic efficiency of our model was significantly better than CEA and ca19-9 (Table 5).

## 4. Discussion

Gastric cancer (GC) is one of the most common malignant gastrointestinal tumors with high morbidity and mortality, affecting the quality of human life [7]. At present, the clinical diagnosis of gastric cancer mainly relies on gastroscopy biopsy. Due to the relatively complex operation of gastroscopy, which also brings great discomfort to patients, it cannot be used as a routine physical examination. It is very important and urgent to search for tumor markers for high sensitivity and specificity. Blood naturally reflects the physiological and pathological state of the body. It is very easy to obtain in clinical practice. The practical application is very convenient and suitable for clinical diagnosis. Both theoretically and clinically, blood is an ideal testing material.

The peptidome refers to the low-molecular-weight proteome of serum protein fragments and peptides and represents an emerging tool for biomarker discovery [8–10]. The content of serum peptides in the blood is relatively low, and the serum peptide which is related to the disease is even lower. Therefore, serum peptides must be concentrated. Otherwise, it cannot be effectively detected. Recently, with the rapid development of biomass spectrometry technology, serum peptides, which used to be considered as biological waste, have become a hot spot in screening tumor blood markers [5, 6, 11–13]. In this study, 122 peptide peaks were obtained through by analysed the serum peptide expression profile in 20 healthy patients and 20 gastric cancer patients with MALDI-TOF MS. The differences of 122 peptide peaks in the two groups were further analyzed, and four serum peptides (No. 26 (2863 Da), No. 29 (2953 Da), No. 16 (1945 Da), and No. 17 (2082 Da)) with different expressions significantly were preliminarily screened. Then, the efficiency of these 4 differentially expressed peptide peaks as diagnostic markers in the diagnosis of gastric cancer was analyzed, and the results showed that the sensitivity and specificity of these 4 differentially expressed peptide peaks in the diagnosis of gastric cancer ranged between 75-90%. Of course, if the reference value is properly adjusted, their sensitivity and specificity of these 4 peptide peaks will increase. According to this characteristic, this study creatively used the ratio of increasing peptide peak expression and decreasing peptide peak expression as the diagnostic index. Based on this principle, a serum peptide-based diagnostic model in gastric cancer was established. An additional 10 healthy and 10 gastric cancer patients were used for external validation of the diagnostic model, and the sensitivity and specificity were up to 90%.

CEA is glycosyl phosphatidyl inositol cell-surface-anchored glycoproteins which are usually at very low levels in healthy adults and upregulated in many kinds of cancers [14, 15]. CEA is usually used as a biomarker for many kinds of cancers, such as colorectal cancer [16, 17] and gastric carcinoma [18, 19]. CA19-9 is a tetrasaccharide that is usually attached to O-glycans on the surface of cells. CA19-9 level is upregulated in many types of cancer, such as colorectal cancer, esophageal cancer, and hepatocellular carcinoma [20, 21]. And CA19-9 is used as a tumor marker in the diagnosis of cancer, particularly in pancreatic cancer [22–25]. Serum CEA and CA19-9 have certain references for the diagnosis of many cancer, including lung cancer, colorectal, and pancreatic cancer [24, 26–29]. Although CEA and CA19-9 were also applied for the diagnosis of gastric cancer, their sensitivity and specificity are not suitable [30–33]. To know whether the diagnostic efficiency of serum peptides in the diagnosis of gastric cancer model is better than that of CEA and CA19-9 currently used in clinical practice, we compared the diagnostic efficiency of CEA and CA19-9 with that of serum peptides in a gastric cancer model. The results showed that the reliability of the serum peptide-based gastric cancer diagnosis model which we constructed was better than CEA and CA19-9. This may be the result of that tumors are diseases caused by multiple gene mutations, and a single protein marker in the diagnosis of tumors cannot reach the sensitivity and specificity required for clinical tumor diagnosis and monitoring.

In this study, the expression pattern of serum peptide was obtained by MB-WCX combined with MALDI-TOF MS, and a number of serum peptides were extracted to establish a serum peptide diagnosis model for gastric cancer for analysis. Compared with the traditional diagnosis of diseases based on a single marker, our results showed that the reliability of the serum peptide-based gastric cancer diagnosis model was better than CEA and CA19-9. This may be the result of that tumors are diseases caused by multiple gene mutations, and one single protein marker in the diagnosis of tumors cannot reach their reliable sensitivity and specificity required for clinical tumor diagnosis and monitoring.

Although the diagnostic model established in this study has high diagnostic efficiency, the sample size is small, and the reliability of the results needs to be further verified. On this basis, the sample size (including healthy people, gastric cancer, and other types of diseases) was further expanded on verification, and the reference value was adjusted to lay a foundation for the final clinical application.

## Figures and Tables

**Table 1:** Sample information of healthy volunteers and advanced gastric cancer patients.

		Healthy volunteers (*n* = 30)	Advanced gastric cancer patients (*n* = 30)
Age	<40	1	2
	40-60	19	17
	>60	10	11

Sex	Male	21	21
	Female	9	9

**Table 2:** External validation results of the diagnostic model.

Group	Accuracy	Gastric cancer	Healthy
Gastric cancer	90.0%	9	1
Healthy	90.0%	1	9

**Table 3:** CEA test in serum of gastric cancer patients and healthy people.

	Gastric cancer	Healthy
0-5 ng/mL	8	25
>5 ng/mL	22	5
Average(ng/mL)	23.3 ± 27.6	1.8 ± 2.3

**Table 4:** CA19-9 test in serum of gastric cancer patients and healthy people.

	Gastric cancer	Healthy
0.1-27 U/mL	11	24
>27 U/mL	19	6
Average(U/mL)	53.7 ± 125.6	11.2 ± 9.3

**Table 5:** Comparison of diagnostic efficiency of serum peptides model with CEA and CA19-9 in gastric cancer.

	Gastric cancer	Healthy
Serum peptides model	90.0%	90.0%
CEA	73.3%	83.3%
CA19-9	63.3%	80.0%

## Data Availability

The datasets generated and analyzed for the current study are available.

## Abbreviations

MB-WCX: Magnetic beads based weak cation exchange
MALDI-TOF MS: Matrix-assisted laser desorption/ionization time of flight mass spectrometry
CEA: Carcinoembryonic antigen
CA19-9: Carbohydrate antigen 19-9.

## Supplementary Materials

Supplementary Materials Supplementary Table 1: list of 122 serum peptides.
