# Supplementary material for: Study on the Diagnosis of Gastric Cancer by Magnetic Beads Extraction and Mass Spectrometry
Source: Biomed Res Int. 2020 Aug 5;2020:2743060. doi: 10.1155/2020/2743060 (PMC7426759; doi:10.1155/2020/2743060)
Supplement: Supplementary Materials — Supplementary Table 1: list of 122 serum peptides. [file 2743060.f1.docx]

**Study on the diagnosis of gastric cancer by magnetic beads extraction and mass spectrometry**

Ning Zhu^1,#^, Xiaoliang Xing^1,#^, Limei Cao^2^, Yingjun Zhang^1^, Ti Zhang^1^, Zhen Li^1^, Fen Zou^3^, Qing Li^1,*^

^1^Hunan Provincial Key Laboratory for Synthetic Biology of Traditional Chinese Medicine, School of public health and laboratory medicine, Hunan University of Medicine, Huaihua 418000, Hunan, P. R. China.

^2^Chenzhou No.1 people′s hospital, Chenzhou 423000, Hunan, P. R. China.

^3^South China University of Technology, Guangzhou 510000, Guangdong, P. R. China.

^#^Contributed equally to this work.

^*^Corresponding author. Correspondence may be addressed to Qing Li. Tel: +86 745 2384439; Email: [180443280@qq.com](mailto:180443280@qq.com)

**Supplementary Table 1 List of 122 serum peptides**

| **Index** | **Mass** | **Index** | **Mass** | **Index** | **Mass** |
| --- | --- | --- | --- | --- | --- |
| 1 | 881.09 | 42 | 4054.21 | 83 | 6003.53 |
| 2 | 1061.49 | 43 | 4072.3 | 84 | 6048.73 |
| 3 | 1105.65 | 44 | 4091.14 | 85 | 6088.25 |
| 4 | 1207.35 | 45 | 4122.31 | 86 | 6331.64 |
| 5 | 1296.96 | 46 | 4150.46 | 87 | 6376.72 |
| 6 | 1330.87 | 47 | 4169.21 | 88 | 6432.29 |
| 7 | 1350.29 | 48 | 4192.79 | 89 | 6453.47 |
| 8 | 1450.32 | 49 | 4210.18 | 90 | 6488.72 |
| 9 | 1466.58 | 50 | 4227.01 | 91 | 6526.23 |
| 10 | 1520.41 | 51 | 4247.11 | 92 | 6563.17 |
| 11 | 1545.9 | 52 | 4266.08 | 93 | 6590.23 |
| 12 | 1570.88 | 53 | 4282.18 | 94 | 6630.78 |
| 13 | 1618.3 | 54 | 4473.45 | 95 | 6665.48 |
| 14 | 1741.82 | 55 | 4529.37 | 96 | 6881.08 |
| 15 | 1887.49 | 56 | 4629.16 | 97 | 6939.37 |
| 16 | 1945.27 | 57 | 4644.17 | 98 | 7019.73 |
| 17 | 2082.46 | 58 | 4672.92 | 99 | 7470.98 |
| 18 | 2093.95 | 59 | 4788.36 | 100 | 7565.59 |
| 19 | 2105.94 | 60 | 4964.09 | 101 | 7599.68 |
| 20 | 2210.6 | 61 | 5064.24 | 102 | 7634.21 |
| 21 | 2559.29 | 62 | 5080.48 | 103 | 7678.19 |
| 22 | 2644.19 | 63 | 5192.81 | 104 | 7765.39 |
| 23 | 2660.89 | 64 | 5247.6 | 105 | 7816.61 |
| 24 | 2672.7 | 65 | 5264.5 | 106 | 7923.34 |
| 25 | 2769.32 | 66 | 5294.26 | 107 | 8141.76 |
| 26 | 2863.23 | 67 | 5318.17 | 108 | 8318.35 |
| 27 | 2881.96 | 68 | 5336.79 | 109 | 8565.67 |
| 28 | 2933 | 69 | 5355.59 | 110 | 8628.86 |
| 29 | 2953.09 | 70 | 5522.28 | 111 | 8655.36 |
| 30 | 2990.99 | 71 | 5541.71 | 112 | 8689.07 |
| 31 | 3192.31 | 72 | 5596.29 | 113 | 8809.43 |
| 32 | 3218.06 | 73 | 5617.19 | 114 | 8866 |
| 33 | 3241.39 | 74 | 5633.78 | 115 | 8919.89 |
| 34 | 3263.19 | 75 | 5713.03 | 116 | 9062.55 |
| 35 | 3277.51 | 76 | 5753.26 | 117 | 9138.78 |
| 36 | 3315.89 | 77 | 5806.16 | 118 | 9176 |
| 37 | 3884.17 | 78 | 5865.53 | 119 | 9289.92 |
| 38 | 3907.13 | 79 | 5886.99 | 120 | 9360.65 |
| 39 | 3935.53 | 80 | 5904.63 | 121 | 9419.86 |
| 40 | 3953.52 | 81 | 5958.61 | 122 | 9510.15 |
| 41 | 3972.9 | 82 | 5984.79 |  |  |
